# Comorbid hypertension and osteoarthritis exacerbates joint remodeling and gait compensations in female rats with milder effects observed in males

**DOI:** 10.1016/j.ocarto.2025.100649

**Published:** 2025-07-16

**Authors:** Carlos J. Cruz, Folly M. Patterson, Janak Gaire, Julianna Gonzalez, Jacob L. Griffith, Angela Philistin, Kyle D. Allen

**Affiliations:** a J. Crayton Pruitt Family Department of Biomedical Engineering, University of Florida, Gainesville, FL, USA; b Pain Research and Intervention Center of Excellence, University of Florida, Gainesville, FL, USA; c Department of Orthopaedic Surgery and Sports Medicine, College of Medicine, University of Florida, Gainesville, FL, USA

**Keywords:** Osteoarthritis, Hypertension, Comorbidity, Neurovascular remodeling, Rat model

## Abstract

**Objective:**

Osteoarthritis (OA) often presents with comorbidities such as hypertension, potentially accelerating OA pathogenesis. We hypothesized that hypertension would exacerbate joint-level pathogenesis and OA-related symptoms in a sex-dependent manner.

**Methods:**

Male and female Spontaneously Hypertensive rats (hypertensive) and Sprague Dawley rats (normotensive) underwent either medial collateral ligament and medial meniscus transection (OA) or skin incision (sham) in the right knee (N ​= ​80; n ​= ​10/group/sex). Symptoms were measured monthly (gait) and bi-weekly (tactile sensitivity) for 8 weeks. Endpoint histology assessed joint-level damage, neurovascular changes, and cytokine levels in synovial fluid.

**Results:**

At endpoint, hypertensive-OA rats had thinner cartilage across the medial tibial plateau than normotensive-OA rats (non-overlapping 95 ​% CI), regardless of sex. While not observed in males, hypertensive-OA females developed larger osteophytes (Q1–Q3 ​= ​0.049–0.124 ​mm^2^) than normotensive-OA females (Q1–Q3 ​= ​0.026–0.047 ​mm^2^, p ​= ​0.02) and ranked higher for CD31^+^ vasculature in the subchondral bone plate (Q1–Q3 ​= ​18.2–21.5) than normotensive-OA females (Q1–Q3 ​= ​1; p ​= ​0.01). Hypertensive-OA females developed a limping gait, shifting stance times from their OA to non-OA limb (stance time imbalance ​= ​1.20 ​± ​1.15 ​%, p ​= ​0.04), offloaded their injured limb quicker (temporal symmetry ​= ​52.5 ​± ​1.4 ​%, p ​< ​0.001), and reduced stride lengths (weeks 4 and 8; hypertensive-OA ​< ​normotensive-OA, p ​< ​0.001). These gait changes were not observed in normotensive-OA females or males, nor in hypertensive-OA males.

**Conclusions:**

Hypertension worsened OA pathogenesis at the joint level, more substantially affecting joint remodeling and gait compensations in females. Our results encourage further investigation into the pathophysiologic drivers linking hypertension and OA, particularly vascular changes and sex differences.

## 1 Introduction

Osteoarthritis (OA) commonly presents with comorbidities such as diabetes [1], hypertension [2], and obesity [3,4]. While hypertension is a component of metabolic syndrome, it may also independently contribute to OA [5,6]. In individuals with obesity and hypertension, OA is more prevalent in those with hypertension even when controlling for body mass index [7]. Thus, characterizing hypertension's role in OA is needed.

Hypertension may influence OA through various physiologic systems. It alters sympathetic activity [[8], [9], [10], [11]] and blood perfusion [6] in the synovium and bone, which may promote cartilage and bone remodeling. Increased intraosseous pressure and vascular invasion at the osteochondral junction may also promote cartilage and bone remodeling [12]. Gut-microbiome dysbiosis [[13], [14], [15]], altered hypothalamic-pituitary-adrenal axis activity [16,17], and autonomic dysfunction [[18], [19], [20]] are all associated with OA; these physiologic systems are also perturbed in hypertension [[21], [22], [23], [24]]. Together, these systemic changes in hypertension may act as drivers of OA.

While hypertension is not a confirmed cause of OA, strong associations exist. Hypertensive rats without OA induction exhibit OA-like changes in cartilage and bone [[25], [26], [27]], and hypertensive rats with surgically induced OA show thinner cartilage than normotensive controls [28]. Clinical reports also associate hypertension with radiographic OA [2,7]. However, studies characterizing the direct impact of hypertension on symptoms and joint-level changes are lacking.

Beyond the joint, characterizing OA symptoms in the context of hypertension is challenging. Hypertensive individuals may experience reduced sensitivity to acute pain but increased sensitivity to chronic pain [29]. Sex differences in OA pain physiology [30] and hypertension [31] may further complicate symptom presentation. Since symptoms alert individuals to seek treatment, understanding these interactions can help clarify the broader disease burden. In our prior work, hypertensive OA rats showed autonomic dysfunction and thinner cartilage compared to normotensive OA rats in a sex-dependent manner [27]. Building on this, we now assess OA symptoms and joint-level changes — including neurovascular changes — in a comorbid rodent model. We hypothesized that comorbid hypertension and OA would exacerbate joint remodeling and gait compensations in a sex-dependent manner.

## 2 Materials and methods

### 2.1 Experimental design

The University of Florida Institutional Animal Care and Use Committee approved all procedures. Spontaneously Hypertensive rats (SHR; 12 weeks of age) served as the hypertension model, and Sprague Dawley rats (12 weeks of age) as normotensive controls. Animal weights are provided in Fig. S2A. Rats were procured from Charles River (Wilmington, MA, USA), acclimated for ≥5 days, and pair-housed with standard bedding on a 12-h light/dark cycle with access to food and water *ad libitum*.

Rats (N ​= ​80) were divided into three cohorts (n ​= ​10/group/sex; Fig. S1) and assigned by random number generator to sham or OA surgery, forming four comorbid groups: normotensive-OA, normotensive-sham, hypertensive-OA, and hypertensive-sham. This design reflects biologically relevant groups while avoiding the complexity of additional interaction terms. Power calculations were performed using a sensitivity analysis in G∗Power (v. 3.1). For monthly gait measurements, n ​= ​10 per group/sex, α ​= ​0.05, β ​= ​0.2 (power ​= ​0.8), and three repeated measures yielded an effect size (f) of 0.20. Five biweekly tactile sensitivity measures yield an effect size (f) of 0.18. These effect sizes indicate that the global statistical model should detect a significant effect if the grouping factor or interaction explains 18–20 ​% or more of the total variance. At endpoint (week 8), synovial fluid and knee joints were collected (detailed methods to follow). Due to postoperative incisional dehiscence, one female normotensive-sham and one male hypertensive-sham were removed, reducing these groups to n ​= ​9. This had a negligible effect on the above-reported sensitivity analysis.

### 2.2 Surgical induction of knee osteoarthritis

Inhalation anesthesia was induced by 3 ​% isoflurane in oxygen, then maintained at 1.5–2%. The right stifle was aseptically prepared, followed by a 1–2 ​cm incision slightly medial to the patella. Blunt dissection exposed the medial collateral ligament. The surgeon was blinded until this point, then informed whether the animal would receive OA or sham surgery. For sham, the incision was closed. For OA, the medial collateral ligament was transected, followed by a radial transection of the medial meniscus at its central aspect (MCLT ​+ ​MMT). Muscle and skin were closed with 4–0 polydioxanone sutures. Postoperative pain was managed with sustained-release buprenorphine (0.05 ​mg/kg) and meloxicam (5 ​mg/kg) for 72 ​h.

### 2.3 Tactile sensitivity

One week before baseline recordings, rats were acclimated to wire-mesh floored cages for 30 ​min daily over three days. Tactile sensitivity was tested using an electronic von Frey device (Bioseb, Pinellas Park, FL, USA), which applies a constant plastic tip with increasing force to the plantar region of the hindfoot until a withdrawal response is detected, as described previously [32]. For each hind paw, three recordings were captured, spaced 5 ​min apart, starting with the ipsilateral hind paw and then the contralateral. Measurements were performed once per animal at each time point, with the median of three recordings reported as the withdrawal threshold (grams-force). To minimize stress or habituation, particularly in hypertensive rats prone to heightened stress responses [33,34], recordings were limited to three per paw.

### 2.4 Spatiotemporal and dynamic gait recordings

One week before baseline, rats were acclimated to our custom gait arena for 30 ​min daily over three days. Arena specifications are detailed here [35] and consists of a 60 ​× ​5 ​× ​10-inch clear acrylic enclosure mounted to an aluminum frame and a 45° angled mirror beneath the arena floor to allow for visualization of the rat's ventral and lateral views. High-speed videography (500 frames/s, Phantom Micro Lab 320 camera) recorded rats walking freely. Spatiotemporal gait variables were calculated using in-house open-source software (https://github.com/OrthoBME/GAITORsuite.git), the GAITOR suite [35,36], an open source MATLAB-based GUI that used color thresholding to track body centroid (velocity), detect foot-strike and toe-off (temporal variables), and isolate paw prints (spatial variables). Simultaneously, peak vertical forces were measured using instrumented floor panels (9317B Kistler force links). Only trials within normal walking velocities (25–75 ​cm/s) and ≥2 gait cycles were included, yielding 3,871 spatiotemporal gait trials and 6,366 dynamic recordings. Gait variables (e.g., stance-time imbalance, spatial/temporal symmetry) are defined in detail here [37].

### 2.5 Joint histopathology

At endpoint, the joint capsule was accessed by creating an opening on the femoral end and lifting the patella and patellar ligament. The patella was removed to facilitate synovial fluid collection in both the tibiofemoral and patellofemoral space. The hind limb was transected at the midshaft of the femur using bone shears, the fibula and muscle were removed, and the femur and tibia were trimmed to isolate the stifle joint. Joints were fixed in 4 ​% paraformaldehyde solution for 48 ​h at 4 ​°C, then decalcified using Immunocal™ (StatLab, McKinney, TX, USA) at 4 ​°C for 12 days, with solution changes every three days. Following decalcification, joints were dehydrated and underwent paraffin processing using an automated tissue processor (Leica VIP6) with graded ethanol (70–100 ​%), xylenes, and paraffin. Frontal 10 ​μm sections of the knee were collected. Slides representing the medial loading region were deparaffinized in xylenes, rehydrated in graded ethanol (100 ​%–50 ​%), stained with hematoxylin/eosin, fast green, and safranin-O, then dehydrated in ethanol (95 ​%–100 ​%) and cleared in xylenes. For synovitis experiments, neighboring slides underwent hematoxylin and eosin staining.

Images of the medial tibial compartment were acquired using an EVOS™ XL Core imaging system (4x objective) and graded using in-house open-source software [38,39]. Cartilage, osteophytes, total epiphyseal area, and bone marrow area were manually segmented using a MATLAB GUI (Fig. S5). A MATLAB script calculated tibial cartilage, osteophyte, subchondral bone, and bone marrow areas based on these segmentations, with measurements converted from pixels to cm. For synovitis, the synovial lining of the medial joint capsule was imaged at 20x. Three blinded graders assigned OARSI scores (0–6) by consesus [40]. Synovitis was similarly scored using Krenn guidelines [41]. Representative images for OARSI (Fig. S6) and synovitis (Fig. S7) scores are provided.

### 2.6 Synovial fluid collection and multiplex cytokine assay

Immediately following euthanasia, synovial fluid from the right stifle was collected and tested for IFN-γ, IL-1β, IL-4, IL-5, IL-6, IL-10, IL-13, CXCL1, and TNF-α. Additional collection and assay details are provided in Supplemental Methods.

### 2.7 Immunofluorescence staining and confocal imaging

Neighboring slides to those selected for safranin-O staining were deparaffinized and rehydrated. Antigen retrieval was performed by placing slides in heated (95 ​°C) citrate buffer (Abcam, ab93678) for 20 ​min using previously described methods [42]. Tissue sections were blocked in a solution of 4 ​% normal goat serum (Abcam, ab7481), 1 ​% bovine serum albumin (Boston Bioproducts, IBB-169), and 0.05 ​% Tween 20 (Fisher Scientific, BP337) in PBS at room temperature for 1 ​h. Sections were then incubated in primary antibodies (1:200 rabbit anti-rat CD31 (Abcam, ab182981) and 1:400 mouse anti-rat neurofilament (NF; Abcam, ab215903)) overnight at 4 ​°C. One section per slide received no primary antibody as a control (Fig. S4). Following three PBS washes, sections were incubated in secondary antibody (1:400 goat anti-rabbit Alexa Fluor 488 (Abcam, ab150077) and 1:400 goat anti-mouse Dylight 633 (Invitrogen, 35,512)) at room temperature for 1 ​h. Slides were cover-slipped using mounting media (Vector Laboratories, H-1400).

Stained sections, focusing on medial tibial plateau lesions, were imaged using a Nikon A1 confocal microscope (20X objective, numerical aperture ​= ​0.5) under constant imaging conditions (488 ​nm: Power ​= ​2 ​%, Gain ​= ​110, Offset ​= ​0; 640 ​nm: Power ​= ​2 ​%, Gain ​= ​150, Offset ​= ​0). In the absence of lesions (e.g., sham group), the corresponding zone (medial 1/3) of the medial tibial plateau was imaged. Z-stacks (step size ​= ​1.8 ​μm) were acquired, and a maximum intensity projected image was created for analysis. To assess NF^+^ or CD31^+^ signal in the subchondral bone plate, a blinded grader ranked images from least to greatest signal. Higher ranks indicate greater NF^+^ or CD31^+^ signal relative to all other rats of the same sex, while a rank of one represents the least signal observed; ties received the same rank.

### 2.8 Statistical analysis

Separate statistical models were generated for males and females. Gait and tactile sensitivity were analyzed using linear mixed effects models, with animal ID as a random factor and group and time as fixed factors (Fig. S10) [43]. To account for strain-related size differences, average foot length was included as a covariate for gait variables (Fig. S2). Normality and equal variance assumptions were verified using quantile-quantile plots and residual plots, respectively. For significant ANOVA results, post-hoc comparisons were made using Tukey's HSD corrections. Non-paired least-squares means were calculated to assess temporal and spatial symmetry (comparison to 50 ​%) and stance time imbalance (comparison to 0 ​%).

For quantitative histology results, normality of model residuals was verified using a quantile-quantile plot. Non-normally distributed data (i.e., OARSI/synovitis scores, CD31/NF ranks) were analyzed using a Kruskal-Wallis test, followed by Dunn's test with Holm's method for multiple comparisons. Cartilage thickness was normalized to each subject's maximum (i.e., % max cartilage thickness) to control for size-related strain differences. Analyses were performed using RStudio (v. 4.0.2). Data analyzed using parametric statistics are reported as mean ​± ​95 ​% CI; non-parametric results as 25th and 75th percentiles (Q1–Q3).

## 3 Results

### 3.1 MCLT ​+ ​MMT resulted in successful induction of OA in males and females

**Fig. 1:**
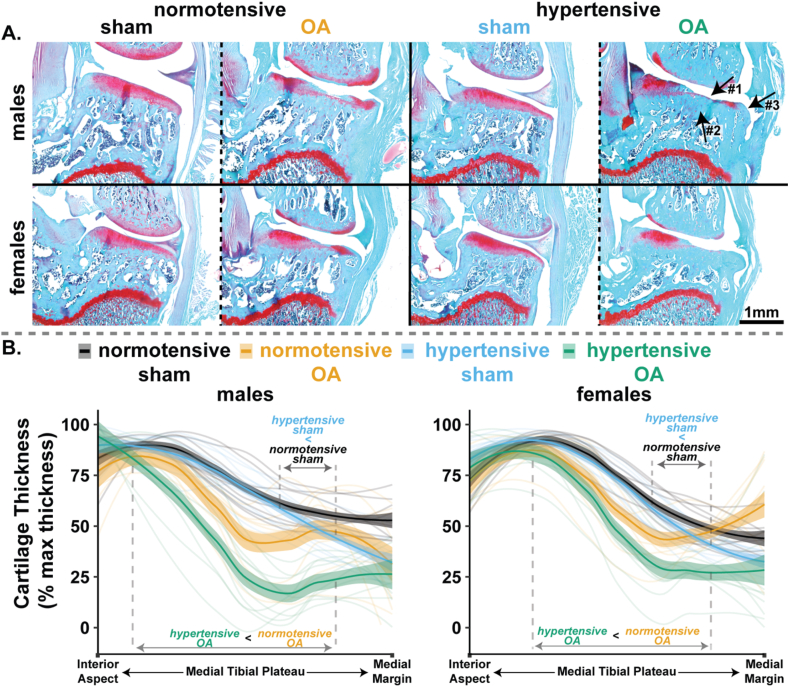
Representative histology and the corresponding cartilage changes. A. Representative safranin-O histology (10 ​μm) imaged at 4X for all groups and sexes. Arrows in top-right show evidence of cartilage loss (#1), subchondral bone thickening (#2), osteophyte (#3). B. Cartilage thickness along the medial tibial plateau normalized to maximum thickness for males (left) and females (right). In B, the x-axis goes from the interior aspect (0 ​%) to the medial margin (100 ​%) of the medial tibial plateau. Data ​= ​mean predicted slope ± 95 ​% CI.

Eight weeks following surgical induction of OA, we observed proteoglycan loss in the cartilage, cartilage lesions, osteophyte formation, and subchondral bone plate thickening in both normotensive-OA and hypertensive-OA rats (arrows on top right of Fig. 1A). These features were not present in their respective sham controls (Fig. 1A). Quantification and comparison of histological features between hypertensive-OA and normotensive-OA animals are provided in the following section.

### 3.2 Hypertension resulted in greater joint remodeling

**Fig. 2:**
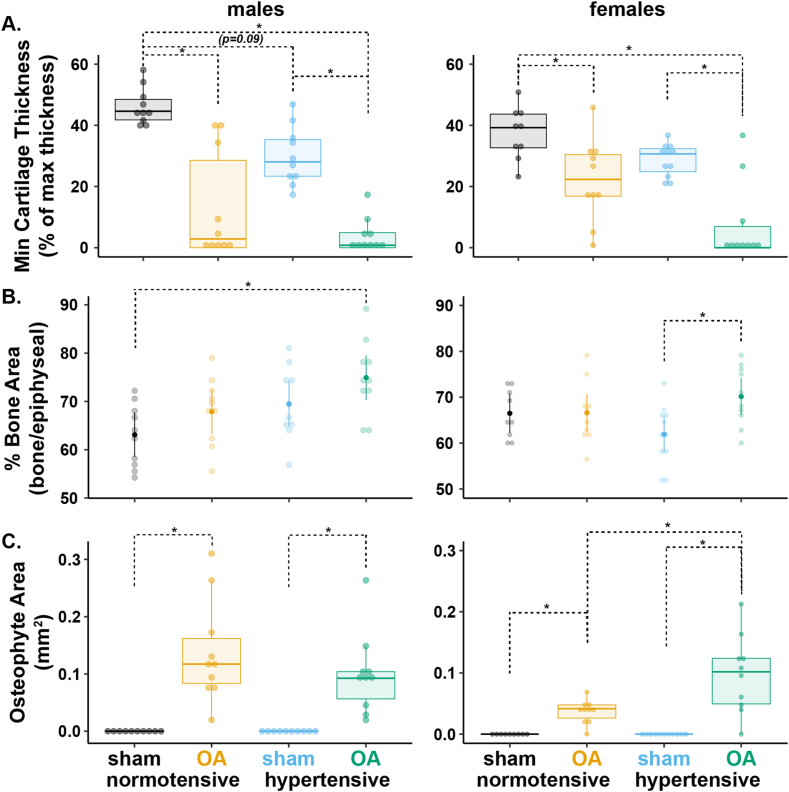
Quantitative histopathology highlighting joint remodeling features for the medial tibial plateau for males (left) and females (right). A. Minimum cartilage thickness normalized to maximum cartilage thickness. B. Bone area ratio of trabecular bone area to total epiphyseal area. C. Osteophyte area in mm^2^. For A and C, data were analyzed using a Kruskal-Wallis test, followed by Dunn's test with Holm's method for multiple comparisons (data ​= ​median and IQR, with whiskers indicating the range of non-extreme data points). For B, an ANOVA was performed, followed by post-hoc comparisons using Tukey's HSD corrections (data ​= ​mean ​± ​95 ​% CI). (∗p ​< ​0.05.).

Spatial evaluation of cartilage changes across the tibial medial plateau showed thinner cartilage in hypertensive-OA compared to normotensive-OA rats (non-overlapping 95 ​% CI in Fig. 1B), regardless of sex. Moreover, hypertensive-sham rats also showed marginally thinner cartilage than normotensive-sham rats near the 1/3 zone of the medial tibial plateau (Fig. 1B). Assessment of cartilage thickness directly at the lesion (non-spatial analysis) showed less cartilage at the lesions of all OA males and females relative to their sham controls (Fig. 2A). Additionally, hypertensive-sham males (Q1–Q3 ​= ​23.3 ​%–35.3 ​%) showed a subtle decrease in minimum cartilage thickness compared to normotensive-sham males (41.7 ​%–48.4 ​%, p ​= ​0.09), although this effect was not observed in females.

Bone area ratio — the ratio of subchondral bone area to total epiphyseal area (subchondral bone and bone marrow) — was not different between normotensive-OA and hypertensive-OA in males or females (Fig. 2B). However, in males, bone area was greater in hypertensive-OA (74.3 ​± ​4.8 ​%) compared to normotensive-sham (61.8 ​± ​4.9 ​%, p ​< ​0.01). In females, only hypertensive-OA (70.1 ​± ​4.1 ​%) showed greater bone area than hypertensive-sham (61.9 ​± ​3.9 ​%, p ​= ​0.02).

Osteophytes were present in all OA rats, regardless of group (Fig. 2C). While osteophyte area did not differ in males, females had greater osteophyte area in hypertensive-OA (Q1–Q3 ​= ​0.049–0.124 ​mm^2^) than normotensive-OA (Q1–Q3 ​= ​0.026–0.047 ​mm^2^, p ​= ​0.02).

**Fig. 3:**
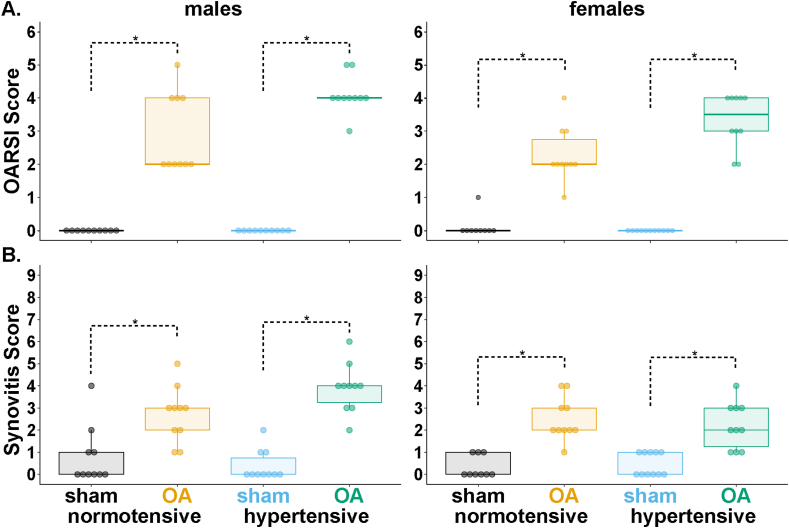
Semi-quantitative histopathology scores for males (left) and females (right). A) OARSI scores of the medial tibial plateau and B) synovitis scores of the synovial lining of the medial joint capsule. Data were analyzed using a Kruskal-Wallis test, followed by Dunn's test with Holm's method for multiple comparisons (data ​= ​median and IQR, with whiskers indicating the range of non-extreme data points). (∗p ​< ​0.05.).

OARSI scores (medial tibial plateau) revealed cartilage damage in all OA rats, regardless of group (Fig. 3A). Male normotensive-OA rats (Q1–Q3 ​= ​2–4) had greater OARSI scores than normotensive-sham (Q1–Q3 ​= ​0, p ​< ​0.01). Male hypertensive-OA rats (Q1–Q3 ​= ​4) also had greater OARSI scores than hypertensive-sham (Q1–Q3 ​= ​0, p ​< ​0.001). Similarly, female normotensive-OA rats (Q1–Q3 ​= ​2.00–2.75) had higher OARSI scores than normotensive-sham (Q1–Q3 ​= ​0, p ​< ​0.01), and female hypertensive-OA rats (Q1–Q3 ​= ​3–4) had higher OARSI scores than hypertensive-sham (Q1–Q3 ​= ​0, p ​< ​0.001). No significant group differences were observed between normotensive-OA and hypertensive-OA groups in either sex for medial or lateral (Fig. S9) OARSI scores.

Synovitis scores for the synovial lining of the medial joint capsule revealed low-grade synovitis in all OA rats, regardless of group (Fig. 3B). No significant differences were observed between normotensive-OA and hypertensive-OA groups in either sex. Male normotensive-OA rats (Q1–Q3 ​= ​2–3) had greater synovitis scores than normotensive-sham (Q1–Q3 ​= ​0–1, p ​= ​0.04). Male hypertensive-OA rats (Q1–Q3 ​= ​3.25–4.00) also had greater synovitis scores than hypertensive-sham (Q1–Q3 ​= ​0.00–0.75, p ​< ​0.001). Similarly, female normotensive-OA rats (Q1–Q3 ​= ​2–3) had higher synovitis scores than normotensive-sham (Q1–Q3 ​= ​0–1, p ​< ​0.001), and female hypertensive-OA rats (Q1–Q3 ​= ​1.25–3.00) had higher synovitis scores than hypertensive-sham (Q1–Q3 ​= ​0–1, p ​< ​0.01).

### 3.3 Hypertensive OA rats ranked higher for vasculature in the subchondral bone plate

**Fig. 4:**
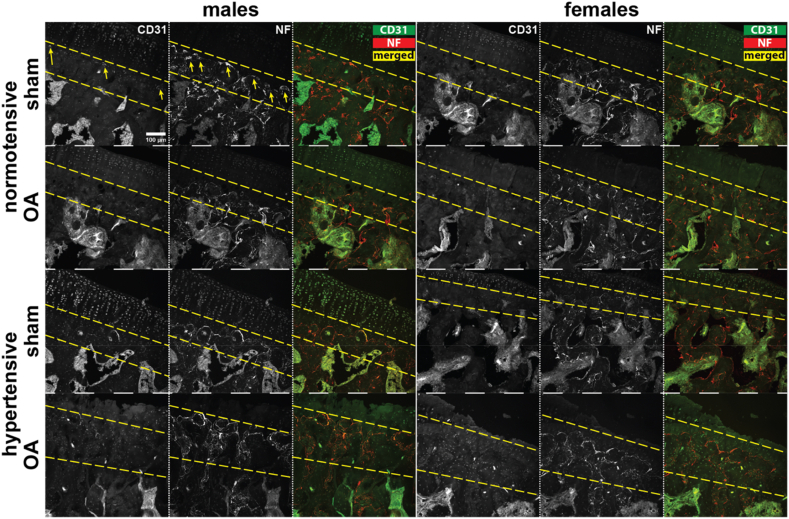
Representative immunofluorescence images (20X) of CD31^+^, NF^+^, and the merged signal in the medial tibial plateau for males (left) and females (right). The approximate region of the subchondral bone plate is outlined with yellow dashed lines for each image (region of interest for CD31 and NF ranks in Fig. 5). Yellow arrows in the top left images indicate example positive signals.

Representative images of CD31^+^ (vasculature) and NF^+^ (nerves) are shown in Fig. 4. Here, CD31^+^ and NF^+^ signal is shown at the subchondral bone plate for all groups.

**Fig. 5:**
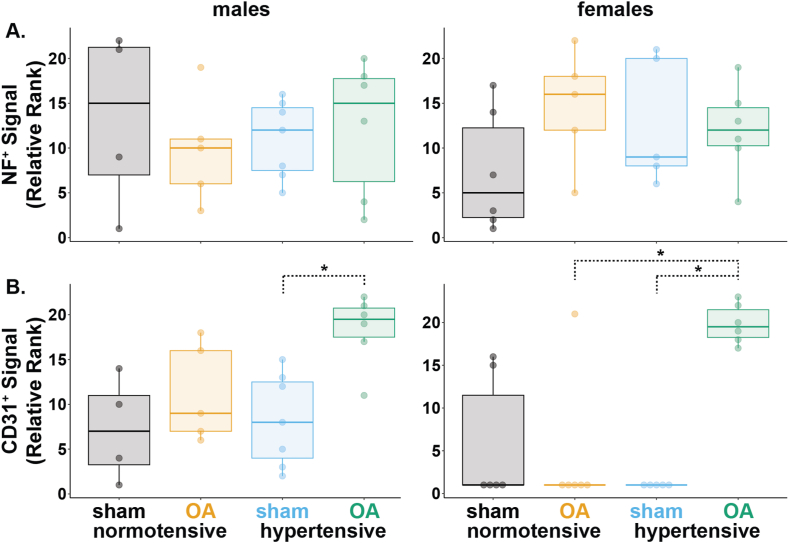
Relative ranks of neurovascular changes in the subchondral bone plate for males (left) and females (right). A. NF^+^ signal intensity. B. CD31^+^ signal intensity. Data were analyzed using a Kruskal-Wallis test, followed by Dunn's test with Holm's method for multiple comparisons (data ​= ​median and IQR, with whiskers indicating the range of non-extreme data points). (∗p ​< ​0.05.).

All groups had similar ranks for NF^+^ fibers in the subchondral bone plate for both males and females (Fig. 5A). Hypertensive-OA females (Q1–Q3 ​= ​18.2–21.5) ranked higher for CD31^+^ vasculature in the subchondral bone plate than normotensive-OA females (Q1–Q3 ​= ​1, p ​= ​0.01; Fig. 5B). For females, all CD31 ranks of 1 indicate no observable CD31 signal. Although hypertensive-OA males (Q1–Q3 ​= ​17.5–20.7) tended to rank higher for CD31^+^ vasculature compared to normotensive-OA males (Q1–Q3 ​= ​7.0–16.0), this comparison was not significantly different (p ​= ​0.27).

### 3.4 Assessing cytokine levels in synovial fluid

Concentrations of IL-10, IL-13, TNF-α, IL-1β, IL-6, IFN-γ, IL-5, CXCL1, and IL-4 were assessed in the synovial fluid for all groups. While most cytokines were below the lower detection limit, detectable levels of TNF-α and CXCL1 were observed in both males and females (Fig. S3). However, no significant group differences were observed for either TNF-α or CXCL1.

### 3.5 Hypertensive females developed an antalgic gait compensation following OA

**Fig. 6:**
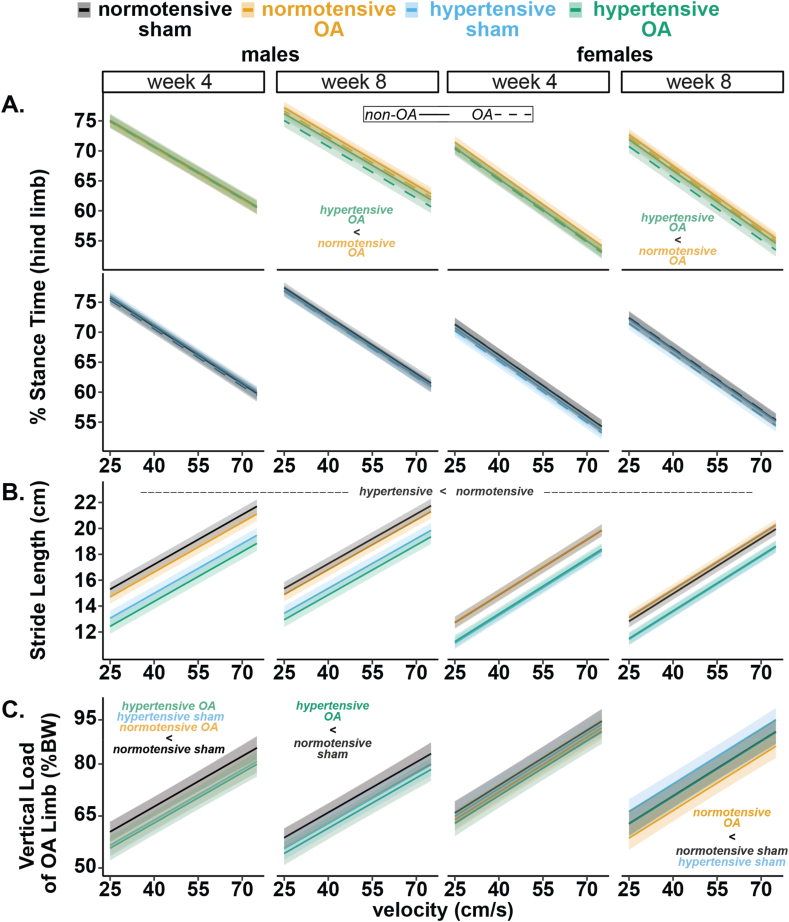
Spatiotemporal and dynamic gait results. Data for males (left) and females (right). A. Percent stance time for the non-OA (solid line) and OA (dashed line) limbs. B. Stride lengths for the hind limbs for all groups. C. OA limb vertical load (peak vertical force, % BW). Data were analyzed using a linear mixed effects model with average foot length included as a covariate to adjust for strain-related size differences. Post-hoc comparisons were made using Tukey's HSD corrections (data ​= ​predicted marginal means ​± ​95 ​% CI). Raw data points omitted for clarity.

No significant stride length differences were observed between OA and sham groups within either sex at weeks 4 or 8 (group main effect, p ​> ​0.05). However, regardless of OA status, hypertensive rats took shorter strides than normotensive rats at all timepoints for males (p ​< ​0.001) and females (p ​< ​0.001; Fig. 6B & Fig. S8B).

**Fig. 7:**
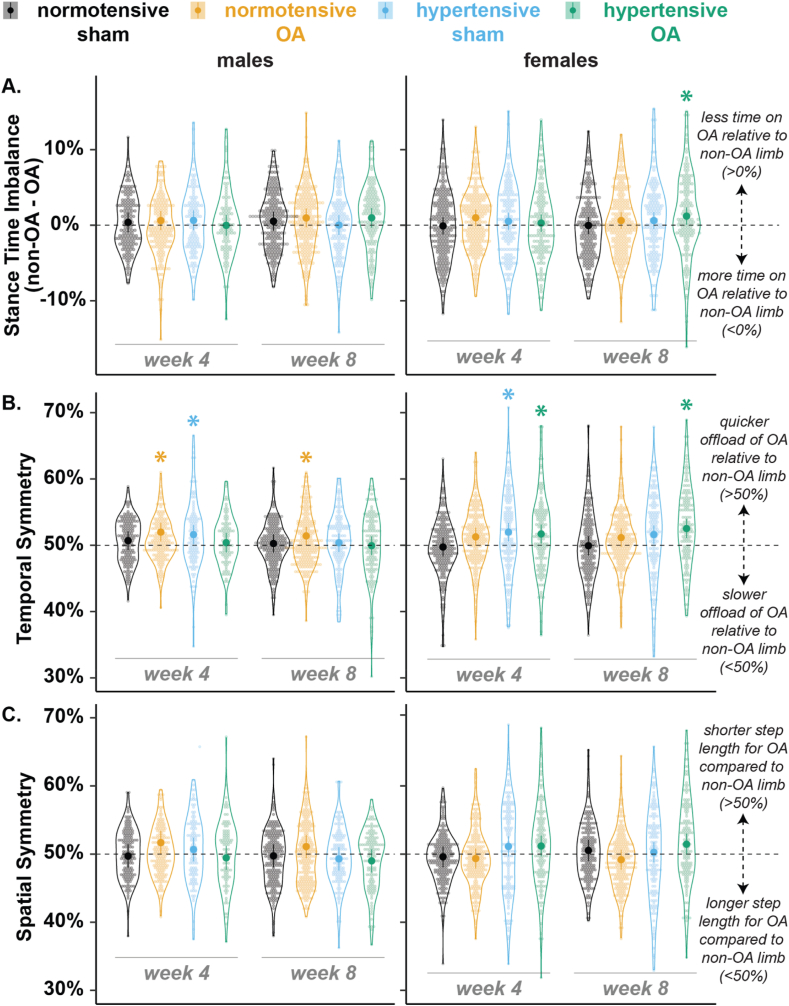
Gait balance and symmetry results for males (left) and females (right). A. Stance time imbalance of the hind limbs. A positive percentage indicates a shift in stance time from the OA to the non-OA limb. B. Temporal symmetry showing where foot strikes occur in time. A positive percentage indicates quicker offloading of the OA limb. C. Spatial symmetry. The dashed lines indicate a symmetric gait pattern. Data were analyzed using a linear mixed effects model, and non-paired least-squares means were calculated to assess temporal and spatial symmetry (comparison to 50 ​%) and stance time imbalance (comparison to 0 ​%) (data ​= ​mean ​± ​95 ​% CI). (Difference from dashed line: ∗p ​< ​0.05).

Percent stance time (i.e., duty factor) — the time between foot-strike and toe-off within a stride time — remained similar for all males and females at week 4 (Fig. 6A). At week 8, hypertensive-OA rats spent less time on their injured limb than normotensive-OA rats for males (p ​= ​0.03) and females (p ​= ​0.04). Stance-time imbalance — defined as stance time of the left (non-OA) minus right (OA) limb of the same animal — remained balanced for all males and females at week 4 (0 ​% dashed line, Fig. 7A). At week 8, stance times remained balanced for males while being imbalanced (stance time shifted from OA to non-OA limb) for hypertensive-OA females (1.20 ​± ​1.15 ​%, p ​= ​0.04).

Temporal symmetry — where a value of 50 ​% indicates that an ipsilateral (OA) foot strike occurred halfway in time between two contralateral (non-OA) foot strikes — was greater than 50 ​% (asymmetric) for normotensive-OA males (51.98 ​± ​1.39 ​%, p ​< ​0.01) and hypertensive-sham (51.61 ​± ​1.43 ​%, p ​= ​0.02) at week 4, while remaining asymmetric for normotensive-OA males (51.41 ​± ​1.36 ​%, p ​= ​0.04) at week 8 (Fig. 7B). For females, at week 4, hypertensive-OA (51.70 ​± ​1.42 ​%, p ​= ​0.02) and hypertensive-sham (51.98 ​± ​1.37 ​%, p ​< ​0.01) were temporally asymmetric, while hypertensive-OA (52.53 ​± ​1.44, p ​< ​0.001) remained asymmetric at week 8. Spatial symmetry — where a value of 50 ​% indicates that an ipsilateral (OA) foot strike occurred halfway between two contralateral (non-OA) foot strikes in space — remained symmetric for all groups at all weeks (Fig. 7C).

Vertical loading of the injured limb was evaluated by calculating peak vertical force and normalizing it to body weight (force in % weight; Fig. 6C). No differences were observed between hypertensive-OA and normotensive-OA for males or females at weeks 4 or 8. However, differences between hypertensive and normotensive rats did arise. At week 4, normotensive-sham males showed significantly greater vertical loads compared to hypertensive-OA males (p ​= ​0.03), and slightly greater vertical loads compared to hypertensive-sham (p ​= ​0.06) and normotensive-OA (p ​= ​0.06). At week 8, normotensive-sham males had greater vertical loads than hypertensive-OA males (p ​= ​0.03). For females, no group differences were observed at week 4. However, at week 8, normotensive-sham females had slightly greater vertical loads than normotensive-OA females (p ​= ​0.06). Additionally, hypertensive-sham females had slightly greater vertical loads than normotensive-OA females (p ​= ​0.06) at week 8.

### 3.6 Hypertension resulted in altered gait patterns at baseline

At baseline, hypertensive males walked with shorter strides (hypertensive-OA ​< ​normotensive-OA, p ​< ​0.001; hypertensive-sham ​< ​normotensive-sham, p ​< ​0.001; Fig. S8B) and a slight decrease in vertical load compared to normotensive males (hypertensive-OA ​< ​normotensive-OA, p ​= ​0.05; Fig. S8C). Hypertensive females also walked with shorter strides (hypertensive-OA ​< ​normotensive-OA, p ​= ​0.01; hypertensive-sham ​< ​normotensive-sham, p ​< ​0.001) and a subtle decrease in vertical load compared to normotensive females (hypertensive-OA ​< ​normotensive-OA, p ​= ​0.07). At baseline, males and females walked with balanced stance times and spatially symmetric gait patterns (dashed control lines, Fig. S8D and S8F). Additionally, all groups were temporally symmetric at baseline except for hypertensive-sham females (51.67 ​± ​1.35 ​%, p ​= ​0.01; Fig. S8E).

### 3.7 Tactile sensitivity remained similar for all groups

**Fig. 8:**
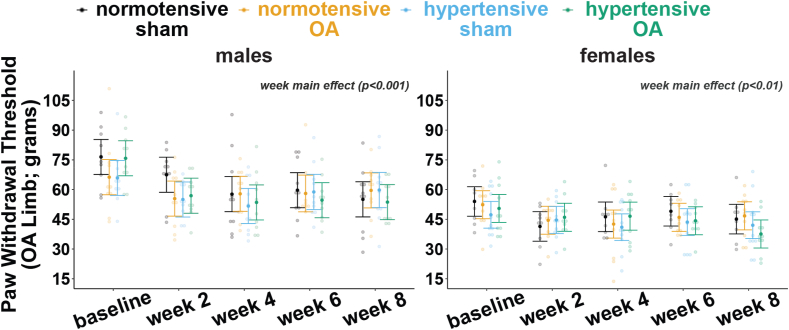
Tactile sensitivity measures for the OA limb for males (left) and females (right). Individual data points correspond to the maximum force applied before paw withdrawal for each individual animal. Data were analyzed using a linear mixed effects model, and post-hoc comparisons were made using Tukey's HSD corrections (data ​= ​predicted marginal means ​± ​95 ​% CI).

Tactile sensitivity was assessed in the hind paw ipsilateral to the OA joint (Fig. 8). Paw withdrawal thresholds decreased across time in males (p ​< ​0.001, week main effect) and females (p ​< ​0.01, week main effect). However, no group differences were observed in either males or females. Group-level estimated marginal means and 95 ​% confidence intervals for each week are provided in Supplemental Tables 1 and 2.

## 4 Discussion

Hypertension is linked to OA through various perturbed physiologic systems. For example, hypertension alters HPA-axis activity [22,23], ANS balance [24], and gut microbiome diversity [21]; perturbations in these systems are associated with OA pathogenesis [[13], [14], [15], [16], [17]]. Peripheral vascular changes also occur in hypertension, which might impact cartilage and bone remodeling [6,12]. However, no prior studies have assessed symptoms, joint remodeling, and neurovascular changes in a comorbid rodent model of hypertension and OA. While the aim of our study was not designed to evaluate whether hypertension causes OA, it did characterize the symptomatic and joint-level consequences of comorbid OA and hypertension in male and female rats. Our experimental design therefore provides new data that builds on the vascular etiology hypothesis of OA [6].

While our use of the earlier OARSI histopathology guidlines [40] confirmed OA development, they did not detect group differences. To better capture structural changes, we applied previously validated tools inspired by the 2010 histopathology guidelines [38,39]. Hypertensive-OA rats showed significantly greater cartilage loss than normotensive OA rats in both sexes. While the eight-week endpoint of the MCLT ​+ ​MMT model coincides with mid-to late-stage OA [44], the extent of cartilage loss observed in hypertensive-OA rats suggests progression beyond this stage. Additionally, hypertensive-OA females exhibited greater joint remodeling, with larger osteophytes compared to normotensive-OA females. Vasculature, assessed using CD31, a marker of endothelial cells [45], was higher in hypertensive-OA females. These results link subchondral vasculature with joint remodeling, potentially driven by abnormal blood perfusion [6,46,47]. This pattern was not observed in males or normotensive rats, suggesting a sex-specific effect in hypertension. Overall, our findings complement a prior meta-analysis showing that radiographic knee OA is more strongly associated with hypertension in women than in men [7]. A possible explanation of our findings in females is the interaction of hypertension and estrogen signaling in hypertensive rats [48], which may also influence vascular remodeling [49] and joint health [50]. Future studies should investigate underlying mechanisms, such as the interaction between sex-hormones and vascular remodeling in hypertension. Additionally, incorporating techniques such as μCT to assess ischemic features (e.g., osteonecrosis) may help clarify the relationship between vascular and structural joint remodeling.

Peripheral nerve remodeling may also contribute to OA pathogenesis in hypertensive individuals [6,51]. While we found no group differences in neurofilament (NF) expression, this doesn't rule out the possibility of peripheral nerve changes at the joint. Since unmyelinated peripheral nerves (e.g., nociceptive Aδ- and C-fibers) express NF at lower concentrations than myelinated nerves, we used a broad NF antibody that detects its light, medium, and heavy chains [52]. Even then, sympathetic and nociceptive nerve fibers, which have been implicated in joint health [9,11,53] and are altered in hypertension [24], are unmyelinated and express lower concentrations of NF. Therefore, future studies should consider more specific antibodies for these targets, such as tyrosine hydroxylase to assess sympathetic innervation or calcitonin gene-related peptide for nociceptors.

Additionally, since joint inflammation promotes peripheral nerve sensitization, we measured cytokine levels in synovial fluid and synovitis histologically. Supporting the validity of our model, low-grade synovitis was found in all OA groups, consistent with the MCLT ​+ ​MMT model, and hypertension did not exacerbate severity. Although synovial cytokines were below the detection threshold using the multiplex MSD assay, our synovitis scoring suggests that inflammation may not be the primary driver of hypertension-related OA changes. Instead, alternative mechanisms — such as disruptions in bone–vascular–cartilage crosstalk — may underlie the structural remodeling observed in hypertensive-OA rats [54]. Clarifying these mechanisms may help define the pathophysiologic pathways linking hypertension and OA.

Despite significant joint remodeling in rats with comorbid hypertension and OA, gait compensations were only observed in females. Hypertensive-OA females walked with an antalgic, or limping, gait compensation, which is typically associated with more pain-like behavior [55]. Interestingly, hypertensive-OA females also developed larger osteophytes, and OA-related symptoms have been shown to coincide more with osteophyte generation [56,57] than cartilage degeneration [58]. Since male OA rats typically show greater symptoms than females [59], hypertension may have reversed that relationship. Additionally, while no changes in tactile sensitivity were observed, the increased sensitivity of the GAITOR suite in detecting mild gait compensations could explain this inconsistency. Overall, this work shows that the relationship between symptoms and certain features of joint remodeling, such as larger osteophytes, may be more direct in females, but less clear in males when OA is comorbid with hypertension.

Independent of OA induction, hypertensive-sham rats showed a subtle decrease in cartilage thickness compared to normotensive-sham rats, suggesting potential pathophysiologic mechanisms driven by hypertension alone. Although mild, these changes align with previous observations of OA-like joint remodeling in naïve hypertensive rats [26]. Since our Spontaneously Hypertensive rats (SHR) weighed less than our normotensive Sprague Dawley rats, body weight or differences in adipose tissue are likely not contributors of this observable cartilage loss. Altered gait patterns were also observed prior to OA induction, with hypertensive rats applying less hind limb vertical load (% weight) and taking shorter strides than normotensive rats while remaining balanced and symmetrical. While the underlying cause of these abnormal gait features is unclear, hypertension is associated with OA-like joint remodeling [25,26] and intervertebral disc degeneration [60]. Although our experimental design could not assess baseline joint pathology, our observations support using hypertensive rats as a model of dysregulated joint health.

Finally, the size differences between strains were a confounder in our study. Sprague Dawley rats being larger than SHRs leads to greater stride lengths. However, our statistical model incorporated foot length as a covariate and normalized vertical load to body weight to control for size and weight differences. While the commonly used normotensive control for SHRs is the Wistar Kyoto rat, it has significant genetic divergence from SHRs [61]. As a result, an appropriate normotensive control for SHRs does not exist. Since the MCLT ​+ ​MMT model of OA has been well characterized in the Sprague Dawley rat and the SHR in our prior work [28], it was selected for our experimental design. However, physiological differences between SHR and SD rats may limit interpretation, as our design cannot delineate whether the observed effects are a consequence of hypertension [6], strain-related metabolic differences potentially driving bone and cartilage changes [62,63], or a combination of both. Ultimately, preclinical models of induced hypertension, such as diet-based models [64] that allow for comparisons within the same strain, could be explored to corroborate findings in SHRs.

In conclusion, hypertension led to significantly greater cartilage loss in both males and females with OA. The alignment of greater joint-level damage, vascular changes, and pain-related behaviors in hypertensive-OA females suggests that hypertension is a significant driver of OA pathology in females. We hypothesize that the increased vascularization may reflect underlying pathophysiologic mechanisms related to abnormal blood flow (e.g., ischemia). Additionally, the abnormal gait patterns and slight reduction in cartilage thickness in hypertensive-sham rats highlight the potential of hypertensive rats as a preclinical model of dysregulated musculoskeletal health. Overall, these results encourage further investigation into mechanisms driving joint remodeling in hypertension, with refined focus on sex differences and vascular changes. While our analyses were limited to within-sex comparisons, they provide an estimate of a potential sex interaction, which may inform future studies specifically powered to assess sex-by-group interactions. In doing so, the development of new therapies or repurposing of existing therapies (e.g., anti-hypertensive drugs) may reduce overall disease burden in the comorbid OA and hypertensive population.

## Author contributions

CJC contributed to the experimental design, animal surgeries, data collection, data analysis and interpretation, and the drafting and revision of the manuscript. FMP contributed to the data collection, data analysis and interpretation, and manuscript revision. Janak Gaire contributed to the histological analysis, data interpretation, and manuscript revision. Julianna Gonzalez contributed to data collection and manuscript revision. JLG contributed to histological analysis, data interpretation, and manuscript revision. AP contributed to data collection and manuscript revision. KDA contributed to the experimental design, data analysis and interpretation, and the drafting and revision of the manuscript.

## Availability of data and materials

The data supporting this study's findings are available from the corresponding author, KDA, upon reasonable request.

## Funding

The National Institute of Arthritis and Musculoskeletal and Skin Diseases of the National Institutes of Health (NIAMS/NIH) supported this study under award numbers R01AR071431 and R01AR071431-03S1. This work was also supported by student fellowships from the Herbert Wertheim College of Engineeringand J. Crayton Pruitt Family Department of Biomedical Engineering at the University of Florida. FMP was supported by the National Institute on Aging (NIA) grant 5T32AG049673 and by NIAMS grant supplement 3UC2AR082196-S2. Beyond providing funds, these sources did not participate in data collection, analysis, interpretation, or decision to submit this publication.

## Declaration of competing interest

KDA is an associate editor for Osteoarthritis and Cartilage. All other authors declare no competing interests.
