# Supplementary material for: Comorbid hypertension and osteoarthritis exacerbates joint remodeling and gait compensations in female rats with milder effects observed in males
Source: Osteoarthr Cartil Open. 2025 Jul 16;7(3):100649. doi: 10.1016/j.ocarto.2025.100649 (PMC12305719; doi:10.1016/j.ocarto.2025.100649)
Supplement: Multimedia component 1 [file mmc1.docx]

**Supplemental Methods**

*Multiplex Cytokine Assay*

Immediately following euthanasia, the joint capsule of the right stifle was accessed by creating an opening on the femoral end and lifting the patella and patellar ligament. A pre-weighed Schirmer strip (Merck Animal Health, Rahway, NJ, USA) was folded, held firmly with a hemostat, and inserted into the joint space for 10-20 seconds. The strip was guided through the joint space and into the suprapatellar bursa, followed by flexing the joint 3–5 times. Schirmer strips were then placed in pre-weighed cryovials, weighed to determine the total collected synovial fluid, and stored at -80°C. Synovial fluid was extracted from Schirmer strips using phosphate-buffered saline (PBS) and shaking at room temperature for 30 minutes. The solution was then eluted by centrifuging at 13,000 RPM for 5 minutes and stored at -80°C until analysis. The total protein concentration of the eluate was determined using a µBCA assay (ThermoFisher, 23227, Waltham, MA). A V-plex Meso Scale Diagnostics (MSD, K15059D, Rockville, MD) assay was used to test for the following targets: IFN-γ, IL-1β, IL-4, IL-5, IL-6, IL-10, IL-13, CXCL1, and TNF-α.
