# Supplementary material for: Comorbid hypertension and osteoarthritis exacerbates joint remodeling and gait compensations in female rats with milder effects observed in males
Source: Osteoarthr Cartil Open. 2025 Jul 16;7(3):100649. doi: 10.1016/j.ocarto.2025.100649 (PMC12305719; doi:10.1016/j.ocarto.2025.100649)
Supplement: Multimedia component 2 [file mmc2.docx]

**Supplementary Figures**

**Supplemental Fig. 1**

**
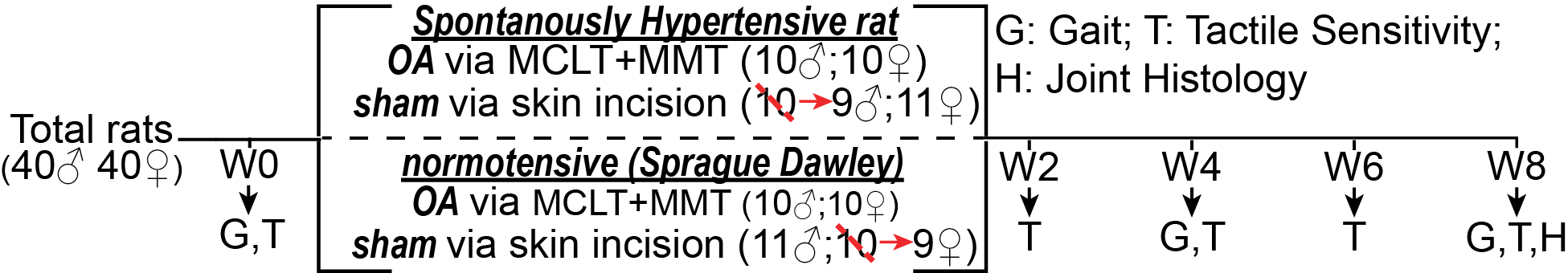
**

**Fig. S1** Summary of experimental design.

**Supplemental Fig. 2**


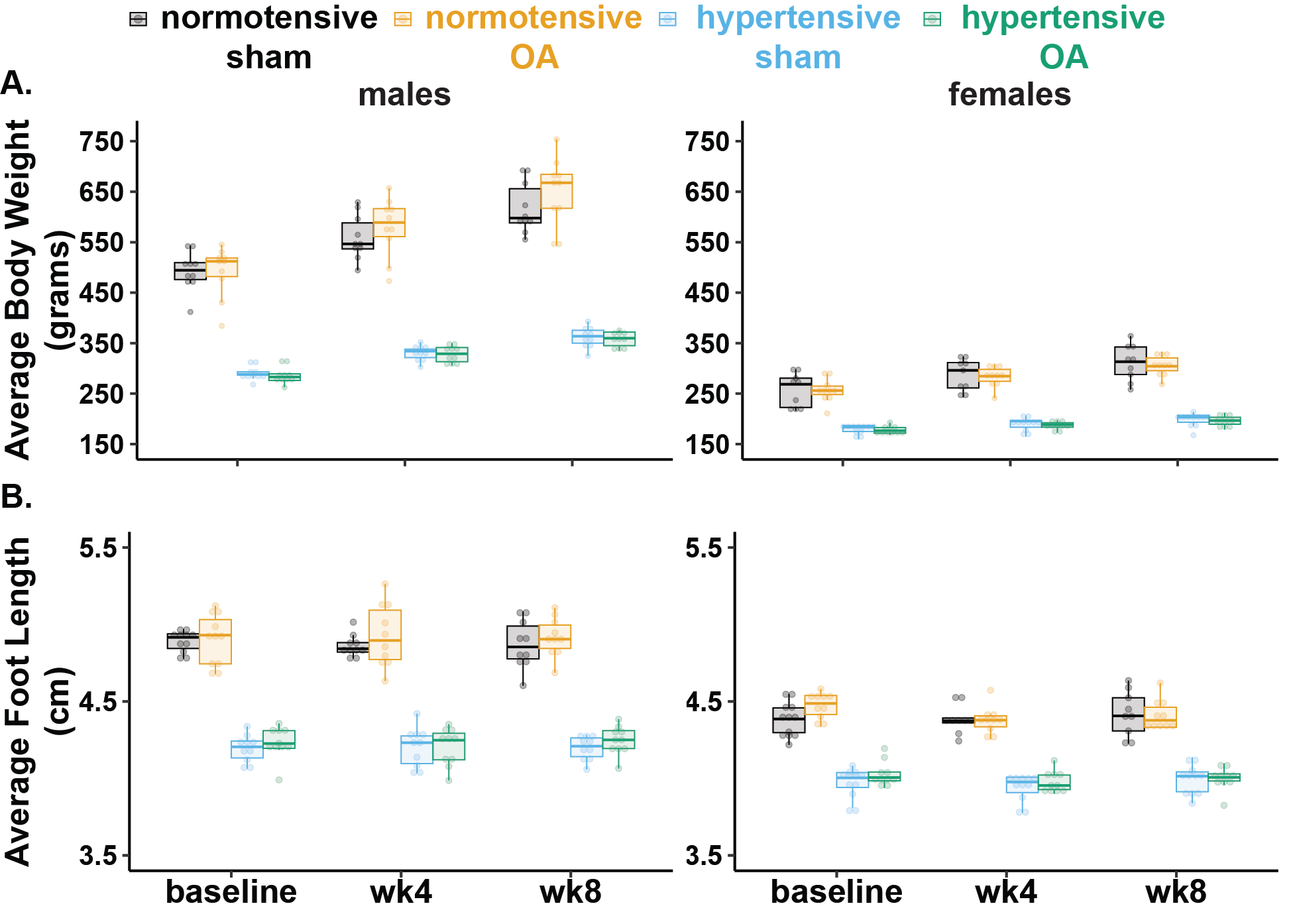


**Fig. S2** Strain differences in body weight and hind foot length. Average body weight (A) and hind foot length (B) for males (left) and females (right). Individual data points correspond with an individual animal's measurement. Box plots represent the median and interquartile range, with whiskers indicating the range of non-extreme data points.

**Supplemental Fig. 3**

**
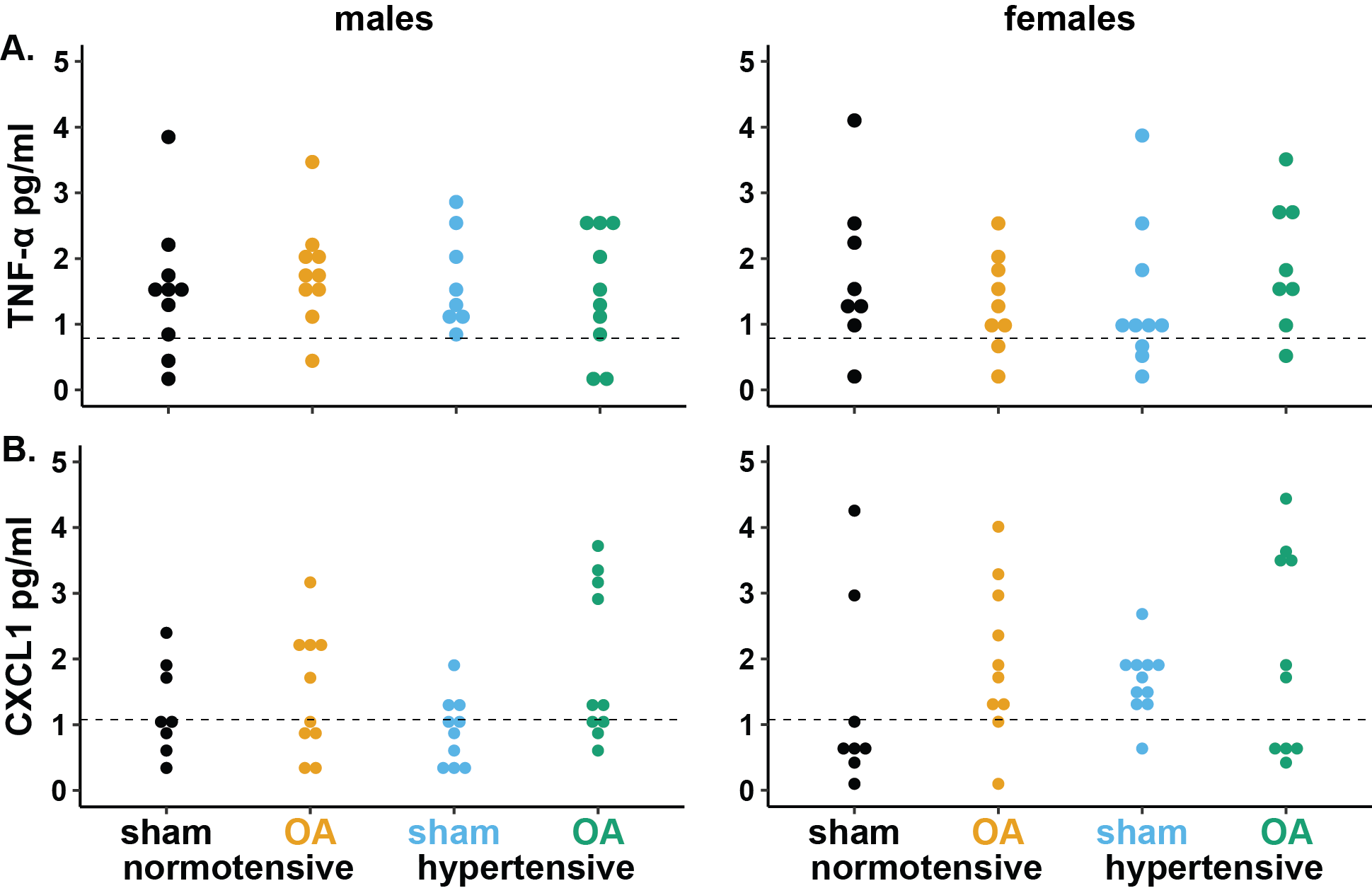
**

**Fig. S3** Cytokine concentration in synovial fluid for TNF-α (A) and CXCL1 (B) in males and females. Dashed lines indicate the lower limit of detection.

**Supplemental Fig. 4**

**Fig. S4** Representative immunofluorescence images (20X) of the medial tibial plateau and subchondral bone plate with no primary antibody (no primary control) for CD31 (left panel; grayscale), NF (middle panel; grayscale), and the merged result (right panel; color).

**Supplementary Fig. 5**


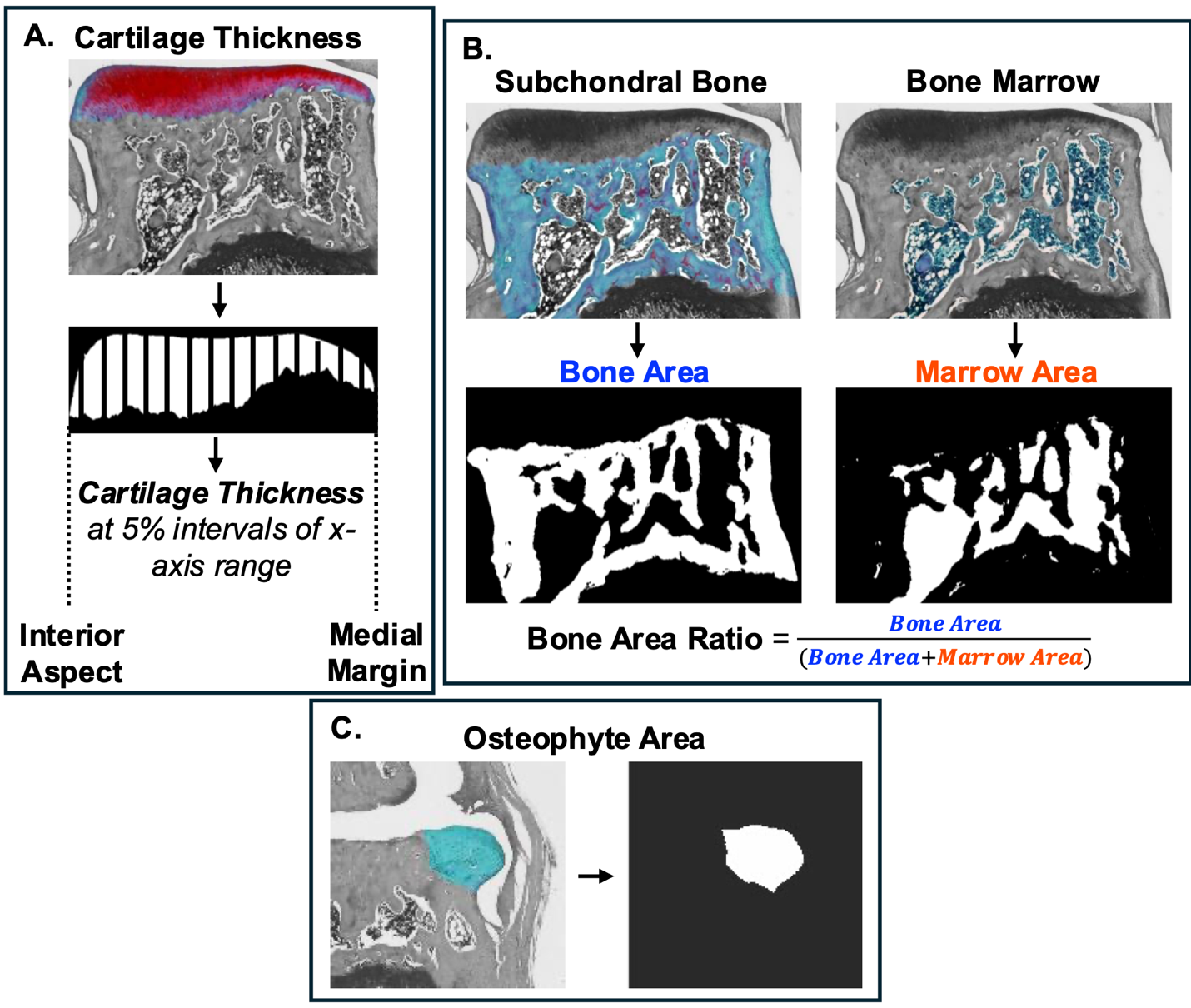


**Fig. S5** Representative segmentations from our quantitative histopathology methodology. A) Cartilage thickness measured at 5% intervals along the medial tibial plateau from the interior aspect to the medial margin. B) Subchondral bone and bone marrow segmentation used to calculate bone area ratio. C) Osteophyte segmentation for osteophyte area quantification.

**Supplemental Fig. 6**

**
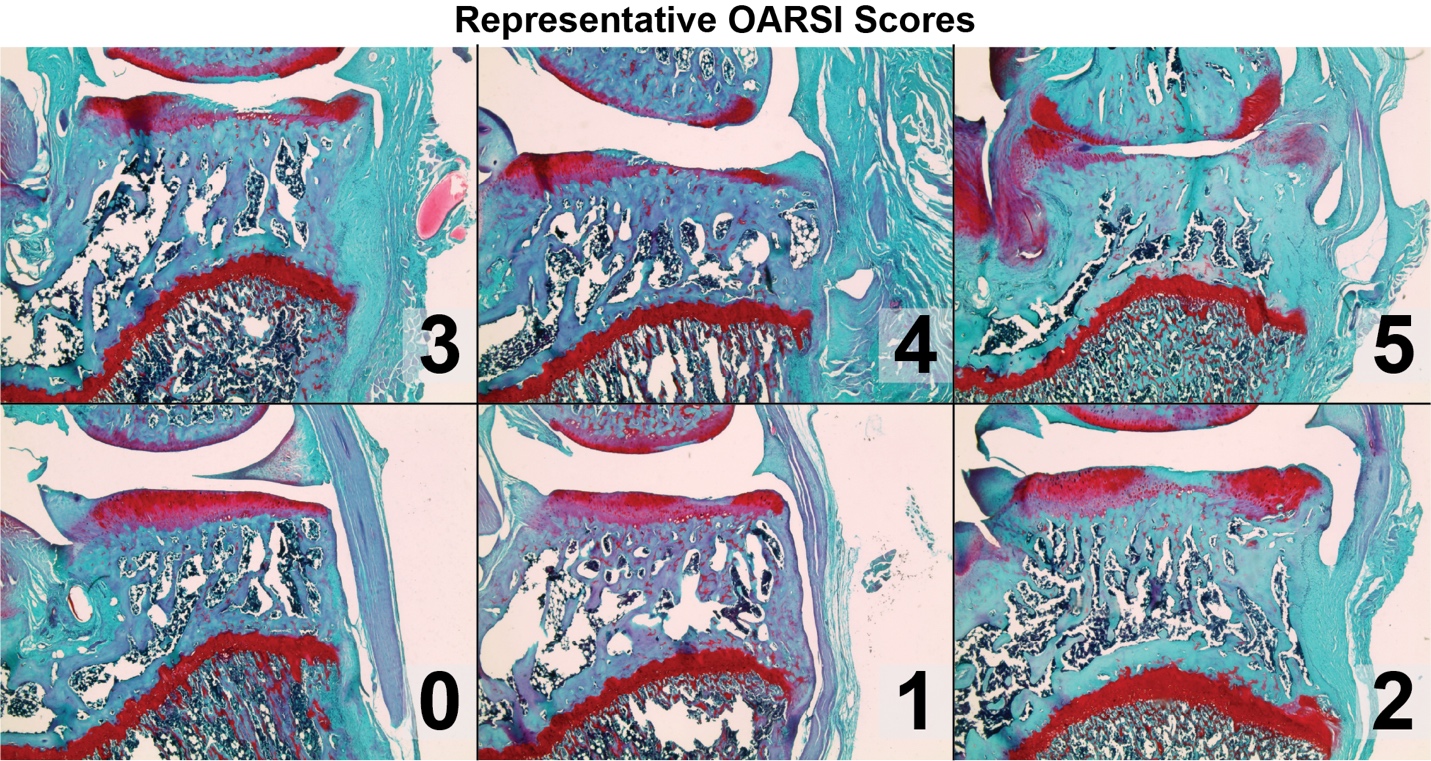
**

**Fig. S6** Representative safranin-O histology (10 μm) imaged at 4× magnification along with their corresponding OARSI scores. No rats received an OARSI score of 6 for this study.

Supplementary Fig. 7


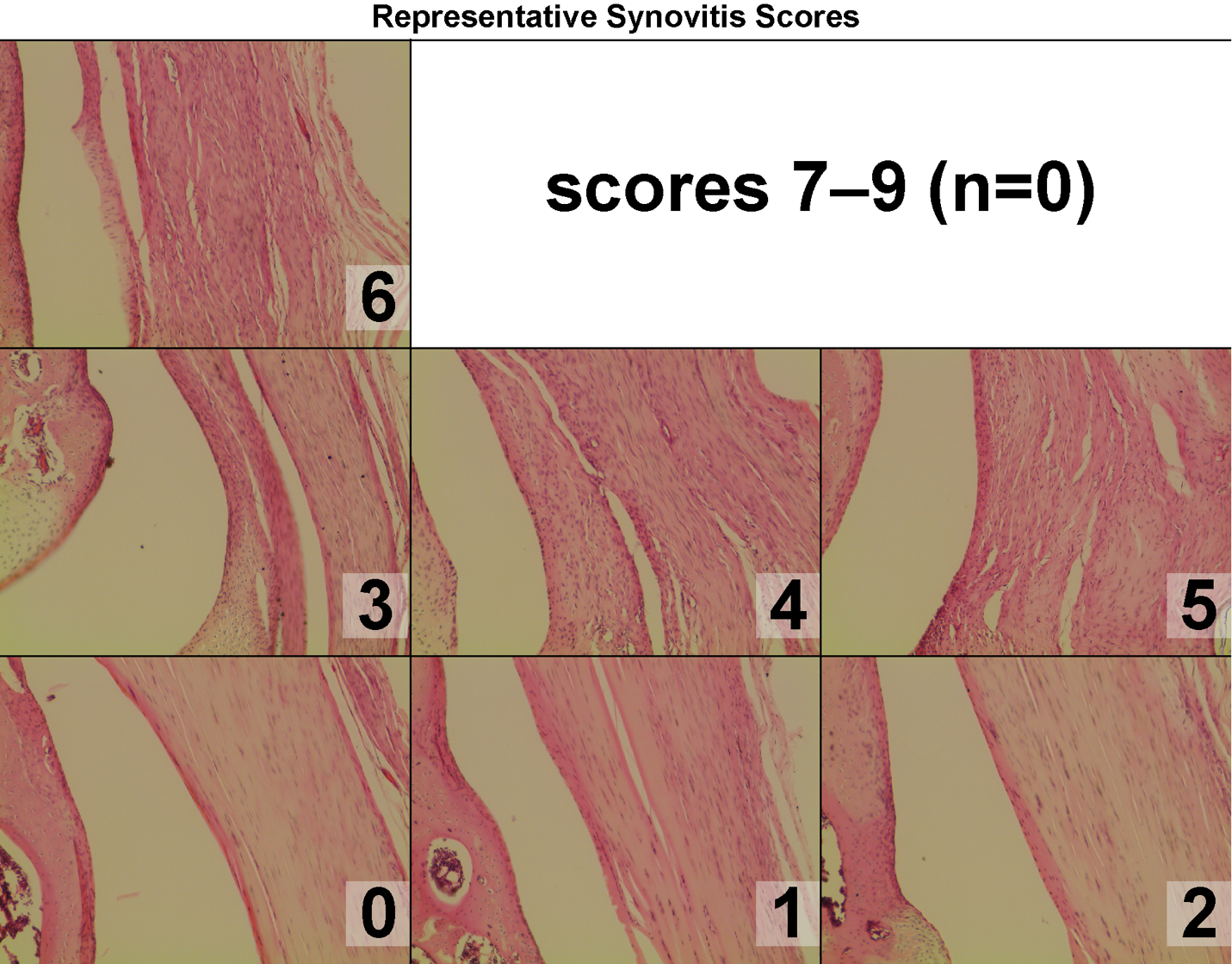


**Fig. S7** Representative hematoxylin and eosin-stained histology sections (10 μm) imaged at 20× magnification, with corresponding synovitis scores based on the Krenn scoring system. Images reflect the synovial lining of the medial joint capsule of the ipsilateral knee. No rats received a synovitis score of 7–9 in this study.

Supplementary Fig. 8


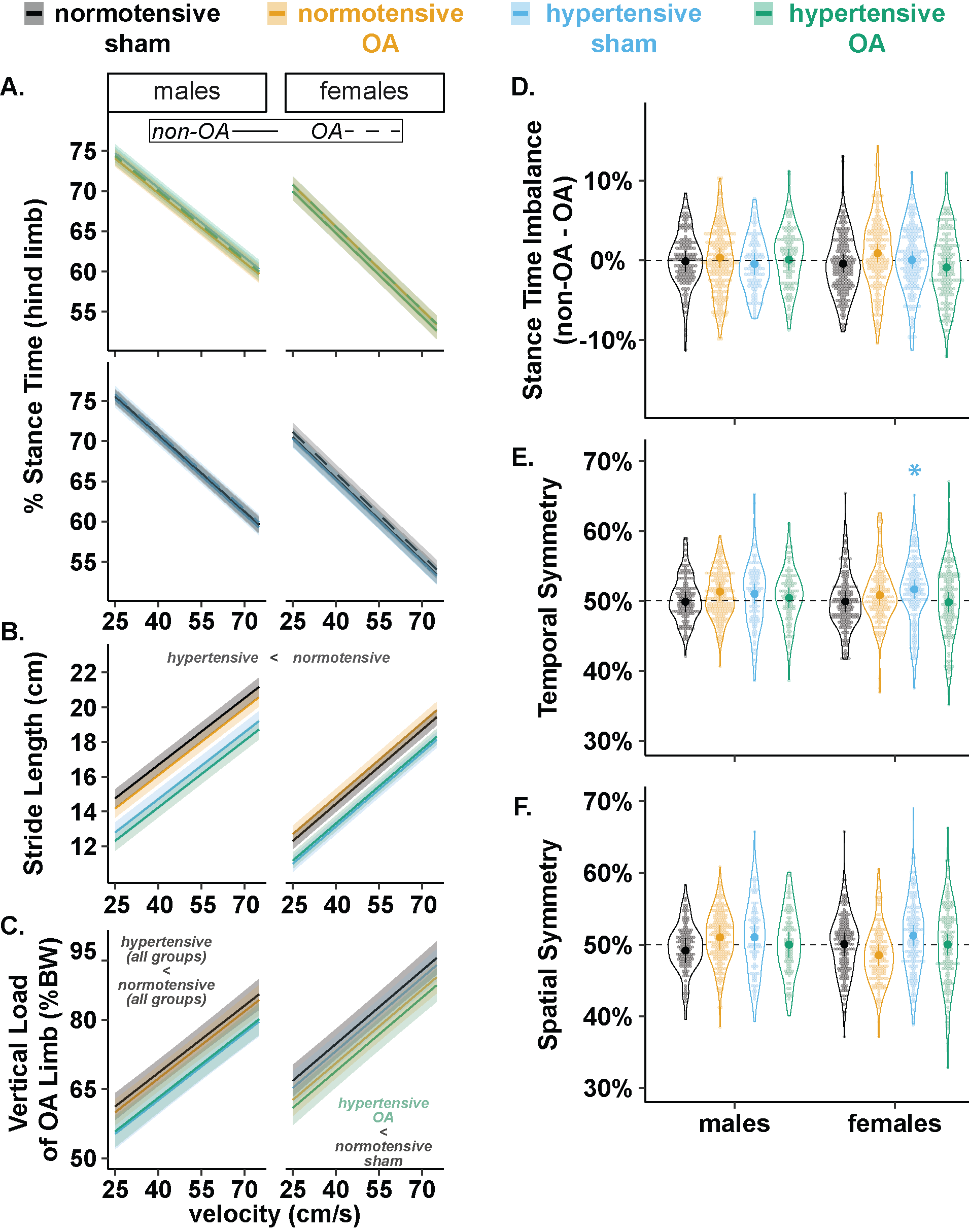
**Fig. S8** Baseline gait measurements for males and females. A. Percent stance time for the non-OA (solid line) and OA (dashed line) limbs. B. Stride lengths for the hind limbs across all groups. C. Vertical load (peak vertical force, % BW) for the OA limb. D. Stance time imbalance of the hind limbs. A positive percentage indicates a shift in stance time from the OA to the non-OA limb. E. Temporal symmetry showing where foot strikes occur in time; a positive percentage indicates quicker offloading of the OA limb. F. Spatial symmetry, with the dashed lines indicating a symmetric gait pattern. All data were analyzed using a linear mixed effects model, with average foot length included as a covariate to adjust for strain-related size differences in stride length. Post-hoc comparisons were made using Tukey's HSD corrections (data = predicted marginal means ± 95% CI). For D–F, non-paired least-squares means were calculated to assess temporal and spatial symmetry (comparison to 50%) and stance time imbalance (comparison to 0%). *p < 0.05.

Supplementary Fig. 9


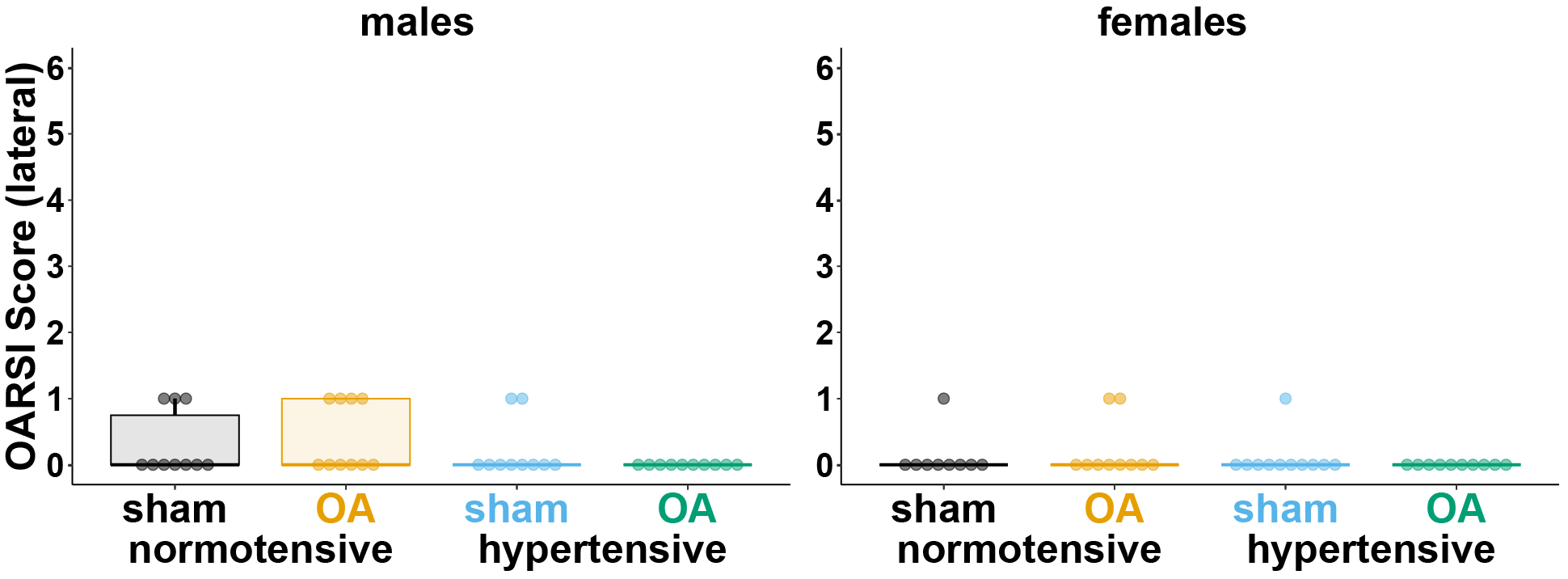


**Fig. S9** OARSI scores of the lateral tibial plateau for males (left) and females (right). Data were analyzed using a Kruskal-Wallis test (data = median and IQR, with whiskers indicating the range of non-extreme data points). (*p < 0.05.)

Supplementary Fig. 10


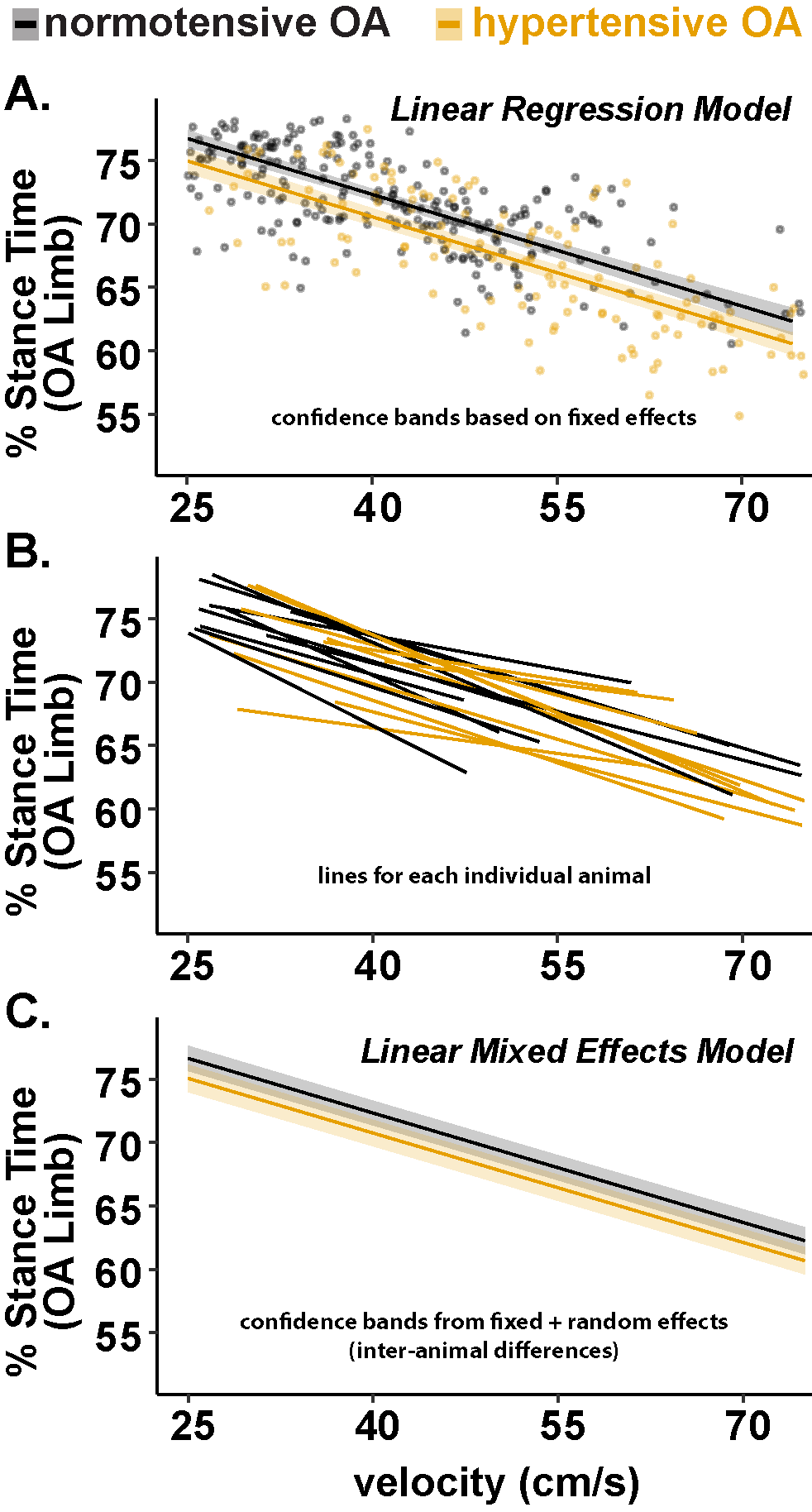


**Fig. S10** Example of gait data (% stance time, OA limb) modeled using linear versus linear mixed-effects approaches. In A, a linear regression model treats each measurement independently and does not account for repeated measurements within each animal. B) illustrates individual animal regression lines, showing inter-animal variability in slopes and intercepts, captured as random effects in linear mixed-effects models. C) shows the linear mixed-effects model confidence bands, which are wider due to inclusion of inter-animal variability, providing more conservative estimates.
