# Supplementary material for: Comorbid hypertension and osteoarthritis exacerbates joint remodeling and gait compensations in female rats with milder effects observed in males
Source: Osteoarthr Cartil Open. 2025 Jul 16;7(3):100649. doi: 10.1016/j.ocarto.2025.100649 (PMC12305719; doi:10.1016/j.ocarto.2025.100649)
Supplement: Multimedia component 3 [file mmc3.docx]

| Supplemental Table 1: Tactile Sensitivity of the OA Limb for Males | | | | | |
| --- | --- | --- | --- | --- | --- |
| group | baseline (mean±95%CI)^1^ | week 2 | week 4 | week 6 | week 8 |
| hypertensive+OA | 75.8 ± 8.9 | 56.9 ± 8.9 | 53.5 ± 8.9 | 54.7 ± 8.9 | 53.7 ± 8.9 |
| hypertensive+sham | 65.9 ± 8.9 | 55.0 ± 8.9 | 51.7 ± 8.9 | 58.8 ± 8.9 | 59.8 ± 8.9 |
| normotensive+OA | 66.3 ± 8.9 | 55.5 ± 8.9 | 57.9 ± 8.9 | 58.1 ± 9.3 | 59.6 ± 8.9 |
| normotensive+sham | 76.5 ± 8.9 | 67.5 ± 8.9 | 57.7 ± 8.9 | 59.7 ± 8.9 | 55.1 ± 8.9 |
| ^1^marginal means and confidence intervals from linear mixed effects model | | | | | |

Supplemental Table 2: Tactile Sensitivity of the OA Limb for Females

| group | baseline (mean±95%CI)^1^ | week 2 | week 4 | week 6 | week 8 |
| --- | --- | --- | --- | --- | --- |
| hypertensive+OA | 50.5 ± 7.1 | 46.0 ± 7.1 | 46.6 ± 7.1 | 44.3 ± 7.1 | 37.6 ± 7.1 |
| hypertensive+sham | 47.3 ± 6.7 | 44.7 ± 6.9 | 41.0 ± 6.7 | 43.6 ± 6.7 | 42.0 ± 6.7 |
| normotensive+OA | 52.4 ± 7.1 | 44.5 ± 7.1 | 42.6 ± 7.1 | 46.0 ± 7.1 | 46.7 ± 7.1 |
| normotensive+sham | 54.0 ± 7.5 | 41.4 ± 7.5 | 46.3 ± 7.5 | 49.1 ± 7.5 | 45.1 ± 7.5 |
| ^1^marginal means and confidence intervals from linear mixed effects model | | | | | |
